# Two-year follow-up of the OptiTrain randomised controlled exercise trial

**DOI:** 10.1007/s10549-019-05204-0

**Published:** 2019-03-26

**Authors:** Kate A. Bolam, Sara Mijwel, Helene Rundqvist, Yvonne Wengström

**Affiliations:** 1 Department of Neurobiology, Care Sciences and Society, Karolinska Institutet, Alfred Nobels allé 23, 14183 Stockholm, Sweden; 2 Cancer Theme, Karolinska University Hospital, Stockholm, Sweden; 3 Department of Cell and Molecular Biology, Karolinska Institutet, Stockholm, Sweden

**Keywords:** Breast cancer, Exercise, Fatigue, Chemotherapy, Physical activity, Long-term effects

## Abstract

**Purpose:**

The aim of this study was to determine if there were any differences in health-related outcomes and physical activity (PA) between the two OptiTrain exercise groups and usual care (UC), 2 years post-baseline.

**Methods:**

The OptiTrain study was a three-arm randomised controlled trial comparing 16 weeks of concurrent aerobic high-intensity interval training (HIIT) and progressive resistance exercise (RT-HIIT) or concurrent HIIT and continuous moderate-intensity aerobic exercise (AT-HIIT) to UC in 206 patients with breast cancer undergoing chemotherapy. Eligible participants were approached 2 years following baseline to assess cancer-related fatigue, quality of life, symptoms, muscle strength, cardiorespiratory fitness, body mass, PA, sedentary behaviour, and sick leave.

**Results:**

The RT-HIIT group reported lower total cancer-related fatigue, (− 1.37, 95% CI − 2.70, − 0.04, ES = − 0.06) and cognitive cancer-related fatigue (− 1.47, 95% CI − 2.75, − 0.18, ES = − 0.28), and had higher lower limb muscle strength (12.09, 95% CI 3.77, 20.40, ES = 0.52) than UC at 2 years. The AT-HIIT group reported lower total symptoms (− 0.23, 95% CI − 0.42, − 0.03, ES = − 0.15), symptom burden (− 0.30, 95% CI − 0.60, − 0.01, ES = − 0.19), and body mass − 2.15 (− 3.71, − 0.60, ES = − 0.28) than UC at 2 years.

**Conclusion:**

At 2 years, the exercise groups were generally experiencing positive differences in cancer-related fatigue (RT-HIIT), symptoms (AT-HIIT), and muscle strength (RT-HIIT) to UC. The findings provide novel evidence that being involved in an exercise program during chemotherapy can have long-term benefits for women with breast cancer, but that strategies are needed to create better pathways to support patients to maintain physical activity levels.

**Trial registration:**

Clinicaltrials.gov registration number: NCT02522260. Trial registered on 9 June 2015. https://clinicaltrials.gov/ct2/show/NCT02522260. Retrospectively registered.

## Background

Regular exercise has shown to be a highly beneficial therapy to manage and improve the physiological and psychosocial health, disease, and treatment-related side effects and symptoms of women with breast cancer [1]. While evidence of the multitude of short-term benefits of exercise for women with breast cancer are generally well established, the long-term effects are less clear. It is essential that we understand the long-term effects of exercise trials if we are to develop and implement meaningful exercise programs with long-term potential following a breast cancer diagnosis and treatment.

A limited number of randomised controlled trials (RCTs) of exercise during chemotherapy in women with breast cancer have followed participants for 1 [2], 4 [3], and 5 years [4, 5]. While these studies add important information to the field of exercise oncology, no study has investigated the long-term effects of two different exercise regimens within the same study. Current international cancer exercise guidelines recommend a structured program including both resistance and aerobic exercise [6]. Despite advances in exercise oncology, we still require stronger and more complete evidence on the optimal exercise prescription for people with cancer according to the FITT principle (frequency, intensity, type, time). Consequently, studies that compare different structured exercise programs within the same study can contribute greatly to our knowledge. In the OptiTrain RCT, a concurrent exercise regimen of high-intensity interval training (HIIT) and high-load resistance training, and a concurrent exercise regimen of moderate-intensity continuous aerobic and high-load resistance training were compared to usual care. The current study provides novel insight into the long-term effects of different exercise modalities within the same exercise trial for women with breast cancer undergoing chemotherapy.

In the OptiTrain study [7], we previously found beneficial short-term effects of the two different supervised 16-week exercise programs (resistance exercise and high-intensity interval training (RT-HIIT), moderate-intensity aerobic exercise and HIIT (AT-HIIT)), on physiological and patient-reported outcomes directly after the intervention [8, 9].

## Methods

### Aim

The aim of this study was to determine if there were any differences in cancer-related fatigue, quality of life, symptoms, muscle strength, cardiorespiratory fitness, body mass, and physical activity levels between the two OptiTrain exercise groups and usual care (UC), 2 years post-baseline.

### Study design and setting

The current study is a 2-year follow-up study of the 16-week OptiTrain randomised controlled exercise trial and was specified in the original OptiTrain protocol [7]. The OptiTrain RCT protocol and the results from the original 16-week exercise intervention [7–9] and 1-year follow-up (Accepted, Journal of Cancer Survivorship, Feb 2019) have been published elsewhere. Briefly, participants were recruited from two oncology clinics in Stockholm, Sweden, from March 2013 to July 2016. Participants were randomised to RT-HIIT, AT-HIIT, or UC.

### Participants

Eligibility criteria were women, 18–70 years of age, diagnosed with stage I–IIIa breast cancer, and scheduled to receive chemotherapy directly.

### Ethics

All procedures performed were in accordance with the ethical standards of the institutional and national research committee (Regional Ethical Review Board in Stockholm, Sweden, registration numbers: 2012/1347-31/1, 2012/1347-31/2, 2013/7632-32, 2014/408-32, 2016/57-32) and with the 1964 Helsinki declaration and its later amendments. The OptiTrain trial has been registered with Clincaltrials.gov (NCT02522260, Optimal Training for Women with Breast Cancer (OptiTrain), http://www.clinicaltrials.gov). Informed consent was obtained from all individual participants included in the study. Two years after the post-baseline, eligible participants were approached and invited to attend an in-clinic assessment session and to complete the online questionnaires.

### Outcome measures

Outcomes were assessed at baseline (1 week before the second chemotherapy session), post-intervention (16 weeks post-baseline), 1 year post-baseline, and 2 years post-baseline. The exceptions to this were objectively measured physical activity (baseline and 2 years only), and pressure pain threshold (baseline, 16 weeks, and 2 years only).

Cancer-related fatigue was assessed by the Swedish version of the revised Piper fatigue Scale (PFS) [10]. Quality of life was assessed by the Swedish version of the European Organisation for Research and Cancer Treatment Quality of Life Questionnaire (EORTC-QLQ-C30) [11]. Symptoms and symptom burden were assessed by the Swedish version of the Memorial Symptom Assessment Scale (MSAS) [12, 13]. Upper and lower body muscle strength were assessed by the isometric mid-thigh pull (Baseline leg dynamometer, Fabrication Enterprises Inc., White Plains, NY, USA) and hand grip tests (JAMAR, SAEHAN corporation, Changwon, S. Korea), respectively [14]. Estimated cardiorespiratory fitness was assessed by the Åstrand-Rhyming submaximal cycle test (Monark 928E, Monark Exercise AB, Vansbro, Sweden). Pressure pain threshold (PPT) was measured bilaterally, with the average of the two measurements calculated (in kilopascals) at the trapezius and gluteus muscles with an electronic algometer (Somedic Sales AB, Hörby, Sweden) [15]. Objectively measured sedentary behaviour and physical activity were assessed by accelerometer (model GT3X ActiGraph® Corp, Pensacola, Florida, USA); the participants were instructed to wear on an elastic belt over their right hip during all waking hours for seven consecutive days. The accelerometer was initialised and data were downloaded using the ActiLife v.6.10.1 software and analysed using validated wear-time specifications and cut-offs for adults [16], which have been published in greater detail in our protocol [7]. Participants also completed a single-item questionnaire asking how much, if any, sick leave they were taking, with five possible options: 0% (not taking any sick leave), 25%, 50%, 75%, or 100% (full-time sick leave).

### Additional measures

Body mass was assessed by calibrated electric scales, and cancer-related and general medical history and participant demographics were recorded by questionnaires. Attendance was calculated as the mean of the individual percentages (number of attended exercise sessions divided by the total number of sessions). Adherence to the exercise regimen was calculated as the number of patients who successfully completed 90% of the exercise sessions according to plan (i.e. intensity and duration), divided by the total number of participants in the intervention groups.

### Intervention

#### Supervised exercise program

The 16-week OptiTrain exercise intervention has been described previously [7, 8]. Briefly, both exercise groups trained twice per week on non-consecutive week days for 16 weeks. Each session was approximately 60 min in duration and was conducted at the exercise clinic at the Karolinska University Hospital. An exercise physiologist or oncology nurse supervised all sessions to ensure safety, correct technique and encourage adherence to the exercise protocols. The RT-HIIT group performed eight resistance exercises of the major muscle groups using machine and free weights. Participants were asked to start with 2 sets of 8–12 repetitions, at an intensity of 70–80% of their estimated one repetition maximum (1-RM). The RT-HIIT sessions concluded with 3 × 3 min bouts of HIIT at a rating of perceived exertion (RPE) of 16–18, with each bout split by one min recovery, on a cycle ergometer. The AT-HIIT group started each session with 20 min of moderate-intensity (RPE 13–15) continuous aerobic exercise followed by the same HIIT regimen as RT-HIIT.

### Follow-up period

Directly after the completion of the 16-week exercise program, participants in the exercise groups were offered a written, physical activity prescription consisting of aerobic and resistance exercises. Participants were also offered a one-on-one exercise counselling session with a professional health educator, and the opportunity to purchase gym memberships, at local commercial gymnasiums throughout Stockholm, at a reduced rate. Additionally, participants were invited to a total of seven sessions that included motivational seminars on a healthy lifestyle, fitness, and training options organised by the research team during the 2-year follow-up period (2014 to 2017).

### Usual care

The UC group received printed written information on general physical activity advice for adults once, directly after baseline testing.

### Statistical analysis and power calculation

The original power calculation was performed with total fatigue, measured by the Piper Fatigue Scale as the primary outcome measure post-intervention (16 weeks). We calculated that we needed a sample size of 65 patients per group, based on an effect size of 0.53 and power = 0.8. From our research group’s experience with participant dropout in previous exercise trials in cancer survivors, we accounted for an attrition rate of ~ 20% and therefore we originally recruited 80 participants into each group.

Baseline demographics were summarised for all participants, and for those who had and had not dropped out of the study 2 years post-baseline. Linear mixed models were used to model the study outcomes at 2 years. Models were adjusted for baseline values of the outcome variable being analysed, menopausal status and tumour receptor status for the primary outcome fatigue measured by the Piper Fatigue scale, and only for the baseline values of the outcome variable being analysed for all other outcomes. Between-group differences were modelled using outcome measurements obtained at 16 weeks (post-intervention), 1 and 2 years post-baseline. Analyses were performed on all participants in the original OptiTrain study with at least one measurement, and only for those who have at least a baseline measurement. Mean differences and 95% confidence intervals (CIs) were accompanied by standardised ES. Statistical significance was set at *p* < 0.05 for all analyses.

## Results

**Fig. 1:**
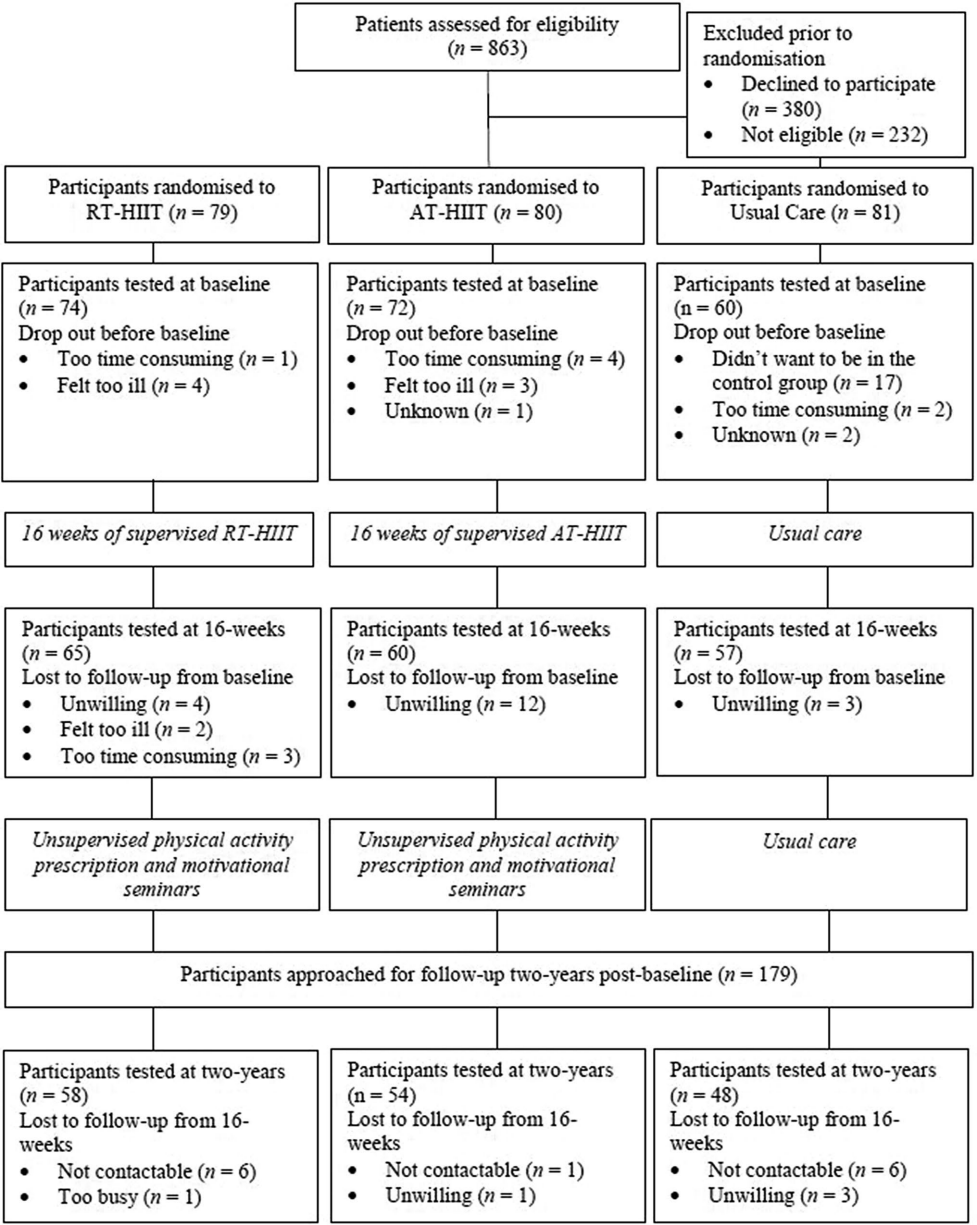
CONSORT diagram: participant flow through the OptiTrain study

Participant flow through the study is shown in Fig. 1. At 2 years post-baseline, a total of 179 participants were eligible and approached to participate in the 2-year post-baseline measurements. At 2 years, 160 participants (66% of those initially randomised and 77% of those who completed baseline testing) completed the questionnaires and 122 participants (51% of those initially randomised and 59% of those who completed baseline testing) completed the in-clinic physiological assessments. Seven participants had died since randomisation and three since the 1-year follow-up. Six participants had recurrences of their cancer between randomisation and the 2-year follow-up. Participants who did not undergo assessments at the 1-year follow-up study were still invited to return for the 2-year assessments (*n* = 12). There were no statistically significant differences in participant characteristics between the three groups at baseline for all participants, and between the three groups for those who completed the 2-year follow-up (Table 1). Additionally, baseline characteristics of all participants and those who completed the 2-year follow-up were comparable (Table 1). Attendance rates for the supervised exercise intervention for participants in the RT-HIIT and AT-HIIT groups were 68% and 63%, respectively. Adherence to the exercise prescription in the supervised exercise intervention was 83% in the RT-HIIT group and 75% in AT-HIIT group. The average attendance for the seven motivational seminars was twenty percent (range 11%–27%).

**Table 1:** Baseline characteristics of all OptiTrain participants and completers 2 years post-baseline

	All participants tested at baseline *n* = 206			Completers 2 years post-baseline *n* = 160		
	RT-HIIT *n* = 74	AT-HIIT *n* = 72	UC *n* = 60	RT-HIIT *n* = 58	AT-HIIT *n* = 54	UC *n* = 48
Age (years)	52.7 ± 10.3	54.4 ± 10.3	52.6 ± 10.2	53.4 ± 10.1	53.9 ± 9.2	54.1 ± 9.6
Body mass (kg)	68.7 ± 11.3	67.7 ± 13.0	69.1 ± 11.0	67.8 ± 10.0	66.6 ± 14.0	69.3 ± 11.4
BMI (kg/m^2^)	25.1 ± 4.3	24.8 ± 4.4	24.6 ± 4.8	25.6 ± 3.8	24.4 ± 3.7	26.0 ± 5.1
MVPA (min/week)	79.8 ± 31.7	70.4 ± 28.9	70.0 ± 36.4	87.0 ± 33.7	78.9 ± 33.6	73.81 ± 28.24
SED (min/day)	523.3 ± 112.7	543.6 ± 109.0	552.8 ± 101.9	542.3 ± 79.0	559.2 ± 81.6	559.2 ± 81.6
Married or partnered	60.6	59.7	69.5	60.3	62.9	66.7
University completed	67.6	64.7	66.0	65.5	68.5	60.4
Current smoker	4.3	5.9	5.2	1.7	5.6	4.2
Postmenopausal	51.4	63.9	61.7	51.7	63.0	58.3
Tumour profile						
Triple negative	14.9	11.0	16.7	10.3	11.1	18.8
HER2+, ER+/−	21.6	30.2	20.0	27.6	31.5	21.3
HER2−, ER+	62.2	58.9	61.6	60.3	58.3	56.2
HER2−, ER−	1.4	0.0	1.7	1.7	0.0	2.0
Chemotherapy received						
Taxane based therapy	40.6	37.0	41.7	35 (22)	34 (21)	27 (17)
Anthracycline based therapy	59.4	63.0	58.3	23 (14)	20 (13)	20 (13)

Continuous variables are presented as mean ± SD, whereas dichotomous or categorical variables are presented as %, RT-HIIT resistance training and high-intensity interval exercise group, AT-HIIT moderate-intensity and high-intensity interval training group, UC usual care, BMI body mass index, MVPA objectively measured moderate- to vigorous-intensity physical activity, SED objectively measured sedentary behaviour, SD standard deviation

### Cancer-related fatigue

At 2 years, there were statistically significant differences between the RT-HIIT and UC, favouring RT-HIIT, for total cancer-related fatigue, (− 1.37, 95% CI − 2.70, − 0.04, ES = − 0.06) and cognitive cancer-related fatigue (− 1.47, 95% CI − 2.75, − 0.18, ES = − 0.28) (Table 2).

**Table 2:** Cancer-related fatigue 2 years post-baseline

		Baseline	16 weeks	2 years		Baseline to 2 years	
		Mean ± SD	Mean ± SD	Mean ± SD		Between-group differences Mean change (95% CI)	ES
Piper fatigue scale							
Total CRF	RT-HIIT	3.14 ± 3.18	3.12 ± 3.03	2.92 ± 2.76	RT-HIIT versus UC	− **1.37 (**− **2.70**, − 0.04)*	− 0.06
	AT-HIIT	2.10 ± 2.63	3.18 ± 2.77	2.34 ± 2.63	AT-HIIT versus UC	− 1.13 (− 2.48, 0.21)	0.11
	UC	2.42 ± 2.90	3.98 ± 3.05	2.37 ± 2.70			
Behaviour/daily life CRF	RT-HIIT	3.14 ± 3.38	3.01 ± 3.31	2.61 ± 2.89	RT-HIIT versus UC	− 1.27 (− 2.65, 0.12)	− 0.36
	AT-HIIT	1.87 ± 2.57	2.98 ± 2.89	2.17 ± 2.70	AT-HIIT versus UC	− 0.68 (− 2.08, 0.72)	− 0.12
	UC	2.13 ± 2.85	4.02 ± 3.29	2.75 ± 2.77			
Emotional/affective CRF	RT-HIIT	3.33 ± 3.43	3.45 ± 3.33	3.30 ± 3.11	RT-HIIT versus UC	− 1.45 (− 2.96, 0.06)	− 0.35
	AT-HIIT	2.37 ± 2.97	3.74 ± 3.23	2.51 ± 2.88	AT-HIIT versus UC	− 1.49 (− 3.02, 0.03)	− 0.33
	UC	2.60 ± 3.15	4.24 ± 3.22	3.74 ± 2.87			
Sensory/physical CRF	RT-HIIT	3.31 ± 3.25	3.27 ± 3.19	3.13 ± 2.89	RT-HIIT versus UC	− 1.28 (− 2.72, 0.15)	− 0.33
	AT-HIIT	2.27 ± 2.87	3.53 ± 3.15	2.50 ± 2.86	AT-HIIT versus UC	− 1.15 (− 2.60, 0.30)	− 0.22
	UC	2.75 ± 3.21	4.29 ± 3.31	3.65 ± 2.96			
Cognitive CRF	RT-HIIT	2.85 ± 2.97	2.82 ± 2.84	2.73 ± 2.65	RT-HIIT versus UC	− **1.47 (**− **2.75**, − 0.18)*	− 0.28
	AT-HIIT	1.88 ± 2.51	2.61 ± 2.39	2.24 ± 2.51	AT-HIIT versus UC	− 1.17 (− 2.47, 0.12)	− 0.13
	UC	2.73 ± 2.79	3.47 ± 2.87	3.43 ± 2.82			

*SD* standard deviation, *CI* confidence intervals, *CRF* cancer-related fatigue, *RT-HIIT* resistance training and high-intensity interval exercise group, *AT-HIIT* moderate-intensity and high-intensity interval training group, *UC* usual care, *ES* effect size, fatigue severity cut-scores: 0 = none, 1–3 = mild, 4–6 = moderate, 7–10 = severe

*p < 0.05

### Symptoms and quality of life

At 2 years, there were statistically significant differences between AT-HIIT and UC, favouring AT-HIIT for total symptoms (− 0.23, 95% CI − 0.42, − 0.03, ES = − 0.15) and symptom burden (− 0.30, 95% CI − 0.60, − 0.01, ES = − 0.19) (Table 3). No statistically significant differences were found between the groups for QoL or the QoL subscales (Table 4).

**Table 3:** Symptoms and symptom burden 2 years post-baseline

		Baseline	16 weeks	2 years		Baseline to 2 years	
		Mean ± SD	Mean ± SD	Mean ± SD		Between-group differences Mean (95% CI)	ES
Memorial symptom assessment scale (MSAS)							
Total symptoms	RT-HIIT	0.74 ± 0.53	0.74 ± 0.50	0.51 ± 0.39	RT-HIIT versus UC	− 0.12 (− 0.30, 0.73)	− 0.08
	AT-HIIT	0.65 ± 0.41	0.76 ± 0.51	0.39 ± 0.31	AT-HIIT versus UC	− **0.23 (**− **0.42**, − 0.03)*	− 0.15
	UC	0.59 ± 0.50	0.85 ± 0.60	0.40 ± 0.33			
Symptom burden	RT-HIIT	0.91 ± 0.72	0.77 ± 0.62	0.62 ± 0.57	RT-HIIT versus UC	− 0.22 (− 0.51, 0.07)	− 0.14
	AT-HIIT	0.75 ± 0.58	0.66 ± 0.59	0.44 ± 0.50	AT-HIIT versus UC	− **0.30 (**− **0.60**, − 0.01)*	− 0.19
	UC	0.71 ± 0.71	0.89 ± 0.67	0.52 ± 0.53			
Physical symptoms	RT-HIIT	0.74 ± 0.59	0.68 ± 0.57	0.35 ± 0.37	RT-HIIT versus UC	− 0.23 (− 0.46, − 0.00)	
	− 0.29						
	AT-HIIT	0.67 ± 0.49	0.75 ± 0.59	0.32 ± 0.33	AT-HIIT versus UC	− 0.22 (− 0.46, 0.01)	− 0.25
	UC	0.52 ± 0.56	0.77 ± 0.62	0.30 ± 0.35			
Psychological symptoms	RT-HIIT	0.97 ± 0.76	1.02 ± 0.77	0.83 ± 0.74	RT-HIIT versus UC	0.02 (− 0.31, 0.34)	0.12
	AT-HIIT	0.81 ± 0.64	0.88 ± 0.73	0.58 ± 0.56	AT-HIIT versus UC	− 0.15 (− 0.48, 0.19)	0.00
	UC	0.81 ± 0.77	1.11 ± 0.83	0.58 ± 0.60			

*SD* standard deviation, *CI* confidence interval, *RT-HIIT* resistance training and high-intensity interval exercise group, *AT-HIIT* moderate-intensity and high-intensity interval training group, *UC* usual care, *ES* effect size

*p < 0.05

**Table 4:** Quality of life 2 years post-baseline

		Baseline	16 weeks	2 years		Baseline to 2 years	
		Mean ± SD	Mean ± SD	Mean ± SD		Between-group differences Mean (95% CI)	ES
European Organisation for Research and Cancer Treatment Quality of Life Questionnaire EORTC-QLQ-C30							
Global/quality of life	RT-HIIT	63.60 ± 24.81	63.85 ± 19.88	71.01 ± 23.80	RT-HIIT versus UC	− 2.22 (− 9.97, 5.53)	0.00
	AT-HIIT	66.67 ± 20.90	63.75 ± 20.29	75.13 ± 18.89	AT-HIIT versus UC	0.93 (− 6.94, 8.81)	0.05
	UC	67.96 ± 21.89	59.52 ± 19.62	75.35 ± 18.87			
Physical functioning	RT-HIIT	89.52 ± 14.62	85.88 ± 16.31	91.48 ± 19.01	RT-HIIT versus UC	2.83 (− 2.89, 8.54)	0.11
	AT-HIIT	89.98 ± 11.41	85.86 ± 15.37	92.45 ± 11.12	AT-HIIT versus UC	3.78 (− 2.03, 9.59)	0.15
	UC	87.55 ± 16.80	76.91 ± 20.22	87.86 ± 17.56			
Emotional functioning	RT-HIIT	67.61 ± 25.71	72.30 ± 22.92	72.30 ± 22.92	RT-HIIT versus UC	1.55 (− 6.20, 9.30)	0.40
	AT-HIIT	74.42 ± 18.94	79.86 ± 16.19	79.86 ± 16.19	AT-HIIT versus UC	6.44 (− 1.42, 14.31)	0.49
	UC	74.48 ± 23.96	69.35 ± 26.26	69.30 ± 26.03			
Role functioning	RT-HIIT	59.55 ± 34.62	70.83 ± 28.27	87.93 ± 23.10	RT-HIIT versus UC	6.19 (− 4.19, 16.57)	0.31
	AT-HIIT	67.61 ± 30.55	71.39 ± 26.59	90.74 ± 16.71	AT-HIIT versus UC	5.96 (− 4.57, 16.49)	0.16
	UC	69.13 ± 28.47	54.46 ± 34.16	87.50 ± 22.13			
Cognitive functioning	RT-HIIT	77.08 ± 25.88	78.19 ± 20.82	76.14 ± 26.88	RT-HIIT versus UC	− 3.56 (− 11.41, 4.29)	− 0.20
	AT-HIIT	81.39 ± 20.60	79.72 ± 19.91	82.39 ± 15.98	AT-HIIT versus UC	− 0.65 (− 8.63, 7.33)	− 0.14
	UC	77.59 ± 25.59	69.94 ± 27.42	81.90 ± 19.98			
Social functioning	RT-HIIT	65.01 ± 29.71	61.76 ± 48.79	80.72 ± 26.65	RT-HIIT versus UC	2.32 (− 8.73, 13.37)	0.11
	AT-HIIT	72.91 ± 24.26	72.78 ± 24.92	87.02 ± 19.06	AT-HIIT versus UC	3.27 (− 7.94,14.48)	0.06
	UC	71.22 ± 30.20	62.20 ± 28.34	83.68 ± 25.84			
Fatigue	RT-HIIT	39.51 ± 29.74	37.58 ± 24.51	24.10 ± 23.17	RT-HIIT versus UC	− 4.77 (− 13.53, 4.00)	− 0.13
	AT-HIIT	35.54 ± 23.28	38.52 ± 24.84	21.76 ± 20.11	AT-HIIT versus UC	− 5.48 (− 14.38, 3.43)	− 0.08
	UC	36.03 ± 27.19	48.81 ± 25.58	24.27 ± 20.21			
Nausea and vomiting	RT-HIIT	13.12 ± 16.43	5.15 ± 10.49	5.78 ± 11.94	RT-HIIT versus UC	− 0.79 (− 6.70, 5.11)	− 0.21
	AT-HIIT	12.99 ± 18.15	5.83 ± 12.21	2.48 ± 9.95	AT-HIIT versus UC	− 3.96 (− 9.96, 2.04)	− 0.38
	UC	8.28 ± 16.94	8.04 ± 21.08	4.52 ± 16.04			
Pain	RT-HIIT	21.62 ± 24.82	21.32 ± 25.08	15.56 ± 19.23	RT-HIIT versus UC	− 7.66 (− 17.03, 1.70)	− 0.39
	AT-HIIT	15.48 ± 22.40	16.95 ± 20.47	15.14 ± 21.04	AT-HIIT versus UC	− 6.56 (− 16.07, 2.95)	− 0.17
	UC	17.32 ± 26.24	27.38 ± 29.89	21.21 ± 26.77			
Dyspnoea	RT-HIIT	24.78 ± 27.29	35.29 ± 29.86	20.65 ± 22.35	RT-HIIT versus UC	0.73 (− 9.61, 11.07)	0.07
	AT-HIIT	22.25 ± 22.46	37.22 ± 28.19	22.17 ± 25.05	AT-HIIT versus UC	3.06 (− 7.69, 12.36)	0.24
	UC	28.07 ± 25.54	44.05 ± 29.89	22.16 ± 23.13			
Insomnia	RT-HIIT	37.01 ± 29.80	31.37 ± 31.48	29.87 ± 32.27	RT-HIIT versus UC	1.25 (− 10.05, 12.55)	0.07
	AT-HIIT	31.85 ± 25.59	26.11 ± 30.74	29.00 ± 25.96	AT-HIIT versus UC	2.76 (− 8.72, 14.24)	0.23
	UC	34.39 ± 31.79	39.88 ± 35.63	25.24 ± 28.42			
Appetite loss	RT-HIIT	19.51 ± 28.89	13.73 ± 24.59	7.17 ± 17.39	RT-HIIT versus UC	− 3.14 (− 12.03, 5.75)	− 0.16
	AT-HIIT	24.50 ± 26.70	20.00 ± 26.89	4.32 ± 13.02	AT-HIIT versus UC	− 8.57 (− 17.64, 0.50)	− 0.48
	UC	14.81 ± 22.94	19.05 ± 27.60	6.60 ± 17.78			
Constipation	RT-HIIT	21.34 ± 27.88	10.78 ± 22.63	10.76 ± 21.39	RT-HIIT versus UC	− 1.00 (− 9.67, 7.67)	− 0.08
	AT-HIIT	21.44 ± 27.09	12.22 ± 21.23	9.23 ± 19.83	AT-HIIT versus UC	− 1.74 (− 10.56, 7.07)	− 0.14
	UC	19.17 ± 27.23	14.88 ± 24.55	8.83 ± 19.82			
Diarrhoea	RT-HIIT	14.33 ± 22.49	8.82 ± 19.63	6.89 ± 19.50	RT-HIIT versus UC	2.32 (− 5.80, 10.45)	0.12
	AT-HIIT	13.15 ± 22.87	18.89 ± 28.37	4.91 ± 11.89	AT-HIIT versus UC	1.21 (− 7.05, 9.47)	0.09
	UC	15.81 ± 23.85	8.33 ± 17.12	5.54 ± 14.29			
Financial difficulties	RT-HIIT	21.44 ± 31.64	25.00 ± 35.21	13.78 ± 31.23	RT-HIIT versus UC	1.87 (− 7.83, 11.57)	0.19
	AT-HIIT	16.54 ± 26.85	22.78 ± 34.98	4.94 ± 15.06	AT-HIIT versus UC	− 3.21 (− 13.07, 6.65)	0.01
	UC	21.62 ± 33.05	19.05 ± 33.55	7.79 ± 22.20			

*SD* standard deviation, *CI* confidence interval, *EORTC-QLQ-C30* European Organisation for Research and Cancer Treatment Quality of Life Questionnaire, *RT-HIIT* resistance training and high-intensity interval exercise group, *AT-HIIT* moderate-intensity and high-intensity interval training group, *UC* usual care, *ES* effect size

*p < 0.05

### Cardiorespiratory fitness, muscle strength, pressure pain threshold, and body mass

No statistically significant differences were found between either exercise group and UC for cardiorespiratory fitness (Table 5). There were statistically significant and clinically meaningful differences between RT-HIIT and UC, favouring RT-HIIT for lower limb muscle strength (12.09, 95% CI 3.77, 20.40, ES = 0.52) but no statistically significant differences between either exercise group for hand grip strength (Table 5). While no statistically significant differences were found for PPT, the RT-HIIT group returned to baseline pain sensitivity levels at the gluteus at 2 years, while the UC group still had elevated pain sensitivity at 2 years (ES = 0.54) (Table 5). Body mass was statistically significant different between AT-HIIT and UC − 2.15 (− 3.71, − 0.60, ES = − 0.28) (Table 5).

**Table 5:** Cardiorespiratory fitness, muscle strength, pain pressure threshold, physical activity, sedentary behaviour, and body mass 2 years post-baseline

		Baseline	16 weeks	2 years		Baseline to 2 years	
		Mean ± SD	Mean ± SD	Mean ± SD		Between-group differences Mean (95% CI)	ES
Estimated VO2peak (L/min)	RT-HIIT	2.25 ± 0.50	2.18 ± 0.57	2.46 ± 0.68	RT-HIIT versus UC	0.09 (− 0.15, 0.32)	0.20
	AT-HIIT	2.10 ± 0.47	2.08 ± 0.49	2.20 ± 0.66	AT-HIIT versus UC	0.03 (− 0.20, 0.26)	− 0.02
	UC	2.19 ± 0.53	1.93 ± 0.53	2.30 ± 0.53			
Estimated VO2peak (ml/kg/min)	RT-HIIT	33.45 ± 7.91	31.70 ± 8.26	35.36 ± 10.14	RT-HIIT versus UC	1.53 (− 2.82, 5.89)	0.14
	AT-HIIT	31.30 ± 6.65	31.36 ± 6.27	33.72 ± 10.56	AT-HIIT versus UC	1.11 (− 3.22, 5.44)	0.22
	UC	32.40 ± 7.79	27.55 ± 6.64	33.24 ± 9.87			
Isometric mid-thigh pull (kg)	RT-HIIT	87.23 ± 29.55	100.24 ± 34.31	105.24 ± 40.11	RT-HIIT versus UC	12.09 (3.77, 20.40)*	0.52
	AT-HIIT	78.35 ± 25.11	88.26 ± 23.02	92.76 ± 29.25	AT-HIIT versus UC	5.53 (− 2.82, 13.88)	0.43
	UC	89.32 ± 25.27	85.81 ± 25.96	92.90 ± 26.01			
Handgrip surgery side (kg)	RT-HIIT	28.40 ± 5.04	29.44 ± 5.27	29.21 ± 6.14	RT-HIIT versus UC	1.23 (− 0.13, 2.59)	0.22
	AT-HIIT	28.44 ± 4.96	28.08 ± 5.29	28.20 ± 5.30	AT-HIIT versus UC	0.75 (− 0.58, 2.07)	0.04
	UC	29.00 ± 6.16	27.72 ± 5.78	28.56 ± 5.18			
Handgrip non-surgery side (kg)	RT-HIIT	27.71 ± 4.93	28.39 ± 5.54	28.30 ± 5.97	RT-HIIT versus UC	0.82 (− 0.67, 2.31)	0.20
	AT-HIIT	27.87 ± 5.44	27.41 ± 5.48	27.61 ± 6.06	AT-HIIT versus UC	0.73 (− 0.73, 2.18)	0.05
	UC	28.47 ± 6.50	27.18 ± 6.33	27.93 ± 5.56			
Pressure pain threshold trapezius	RT-HIIT	419.19 ± 142.46	448.40 ± 149.84	401.26 ± 170.48	RT-HIIT versus UC	33.03 (− 20.81, 86.86)	0.28
	AT-HIIT	402.29 ± 154.23	388.00 ± 122.79	356.71 ± 152.84	AT-HIIT versus UC	7.37 (− 44.24, 58.98)	0.08
	UC	401.54 ± 134.07	366.05 ± 124.57	344.50 ± 133.38			
Pressure pain threshold gluteus	RT-HIIT	420.54 ± 144.60	441.00 ± 134.38	422.57 ± 206.57	RT-HIIT versus UC	37.38 (− 11.85, 106.61)	0.54
	AT-HIIT	422.44 ± 196.89	413.24 ± 145.84	356.30 ± 152.83	AT-HIIT versus UC	0.91 (− 55.85, 57.67)	0.05
	UC	429.03 ± 142.97	372.63 ± 140.54	353.82 ± 134.40			
MVPA (min/week)	RT-HIIT	79.83 ± 31.67	–	86.99 ± 33.73	RT-HIIT versus UC	0.12 (− 11.42, 11.67)	0.10
	AT-HIIT	70.40 ± 28.88	–	78.94 ± 33.59	AT-HIIT versus UC	5.86 (− 5.19, 16.92)	0.15
	UC	70.04 ± 36.42	–	73.81 ± 28.24			
SED (min/day)	RT-HIIT	523.26 ± 112.68	–	542.26 ± 79.03	RT-HIIT versus UC	28.23 (− 7.21, 63.66)	0.14
	AT-HIIT	543.61 ± 109.04	–	559.23 ± 81.56	AT-HIIT versus UC	25.55 (-8.53, 59.62)	0.11
	UC	552.83 ± 101.93	–	559.23 ± 81.56			
Body mass (kg)	RT-HIIT	68.65 ± 11.34	69.47 ± 10.56	70.46 ± 10.03	RT-HIIT versus UC	− 0.70 (− 2.28, 0.88)	− 0.03
	AT-HIIT	67.66 ± 13.00	67.34 ± 14.45	66.41 ± 10.81	AT-HIIT versus UC	− **2.15 (**− **3.71**, − 0.60)*	− 0.28
	UC	68.82 ± 11.05	71.01 ± 11.66	70.94 ± 12.73			

*SD* standard deviation, *CI* confidence interval, *RT-HIIT* resistance training and high-intensity interval exercise group, *AT-HIIT* moderate-intensity and high-intensity interval training group, *UC* usual care, *MVPA* objectively measured moderate- to vigorous-intensity physical activity, *SED* objectively measured sedentary behaviour

*p < 0.05

### Sedentary behaviour and physical activity

No statistically significant differences were found between either exercise group and UC for minutes of sedentary behaviour or moderate to vigorous physical activity (Table 5).

### Sick leave

For those eligible (i.e. not retired), there were no significant differences between the groups for sick leave with 86%, 89%, and 89% reported that they were not on any sick leave, and 14%, 11%, and 11% reported being on some level of sick leave (either 25%, 50%, 75%, or fulltime) for RT-HIIT, AT-HIIT, and UC, respectively.

## Discussion

The current study examined the long-term effects of the OptiTrain exercise intervention on cancer-related fatigue, symptoms, quality of life, cardiorespiratory fitness, muscle strength, pressure pain threshold, sedentary behaviour and physical activity, and body mass. Generally, those in the exercise groups were still experiencing favourable differences in a number of physiological and patient-reported outcomes in comparison to UC. Participants in the RT-HIIT group reported significantly lower total and cognitive cancer-related fatigue, and had higher leg muscle strength (reaching clinically meaningful effect sizes) than UC at 2 years. However, the AT-HIIT group reported significantly lower total symptoms and symptom burden, and body mass at 2 years post-baseline. There were no significant differences between the groups for minutes of MVPA or sedentary behaviour at 2 years.

Participants in the RT-HIIT group reported statistically significant differences in total and cognitive cancer-related fatigue than the usual care group. While both effect sizes are small and participants generally experienced low levels of fatigue during the study (a score of < 4), these findings are important to show the positive effect exercise can have even for individuals experiencing lower levels of fatigue. At 1 year, both exercise groups reported favourable and significant differences in total cancer-related fatigue, affective/emotional fatigue, and behaviour/daily life fatigue. Additionally, AT-HIIT rather than the RT-HIIT reported favourable differences in cognitive fatigue at 1 year. In this 2-year study, that only the RT-HIIT group managed to avoid an increase in total or cognitive fatigue during chemotherapy and in the follow-up period could possibly be due to the combined effects of both resistance and aerobic exercise on fatigue. Witlox and colleagues found similarly positive yet, non-significant effects of an 18-week program of concurrent aerobic and resistance exercise on physical fatigue in patients with breast and colon cancer in the Physical Activity during Cancer Treatment (PACT) study [3]. The implications of the findings from our study are that those who were prescribed a concurrent regimen of HIIT and resistance exercise were still experiencing positive effects on one of the most commonly reported and distressing side effects, fatigue [17], more than a year after the exercise program had finished.

AT-HIIT, but not RT-HIIT, reported significantly fewer overall symptoms and less symptom burden than UC at 2 years. While producing modest effect sizes, it is remarkable that differences in symptoms between the groups can still be distinguished despite finishing treatment 20 months prior to the 2-year assessment. Again, the effects at 2 years are smaller than the 1-year follow-up, where both exercise groups reported significantly fewer total and physical symptoms than UC. However, the finding at 2 years that it was only AT-HIIT that reported lower symptom burden was also found at 1 year. This is the first study to show long-term differences (longer than a year following the end of the intervention) in symptoms following an exercise intervention for women with breast cancer. Additionally, the questionnaire used in this study was a symptom-specific questionnaire, which allows analyses of the extent to which the participant was experiencing symptoms, which type of symptoms they were experiencing, and the burden of these symptoms.

At 2 years, a clinically and statistically significant difference in lower body muscle strength remained, but only for the RT-HIIT group. To the best of our knowledge, this is the first study to demonstrate favourable differences in muscle strength at the 2-year follow-up of an exercise trial during chemotherapy for women with breast cancer. This finding is clinically important because low muscle strength can be a predictor of cancer-related fatigue in older breast cancer survivors [18] and in healthy older adults muscle strength has been identified as a strong predictor of all-cause mortality [19]. Additionally, muscle strength has overtaken muscle mass as the principal determinant of sarcopenia, which is associated with increased falls, impaired independence, lower quality of life, and premature death [20]. This 2-year result is in contrast to the 1-year follow-up, where both exercise groups still had favourable significant differences in muscle strength to the control group. We speculate that this difference may be in part due to positive long-term exercise adherence effects (creating of habits) of being involved in a supervised resistance exercise program, and the potential knowledge gained during the intervention.

The current study adds novel information to the field of exercise oncology in that it is the first follow-up studies of a RCT, and the longest follow-up of any trial in women with breast cancer, that used accelerometers to measure physical activity and sedentary behaviour, which eliminate the inherit issues of over reporting PA and underreporting sedentary behaviour associated with self-report PA questionnaires [21]. While both exercise groups increased the MVPA from baseline, these differences were not statistically significant from the small increase also measured in the usual care group. Findings from our OptiTrain trial are line with conclusions from the RCT from Schmidt and colleagues who found that physical activity levels generally returned to baseline levels again 12 months following the exercise intervention [2]. The PACT RCT by Witlox et al. found that self-reported levels of MVPA decreased during the exercise intervention/chemotherapy, and had not returned to baseline levels at 36 weeks, but that those in the exercise group did return to baseline levels of MVPA at 4 years [3]. A positive result from the OptiTrain study is that participants in the exercise groups increased their MVPA slightly at 2 years, albeit not statistically significantly, despite undergoing chemotherapy and being without supervised exercise for approximately 20 months prior to the 2-year follow-up. Despite the 16-week intervention and motivational seminars, only three participants (spread evenly across the three groups) reached the recommended 150 min of MVPA per week at 2 years [6]. While it is promising that the three groups did not decrease their min of MVPA from baseline to 2 years, the low number of participants reaching recommended PA levels is cause for concern and this group of patients may require additional support, than was provided in this study, to maintain PA levels following a supervised exercise intervention or program. This conclusion in itself is not novel; this is, however, the first study to provide evidence of this issue using objectively measured physical activity in patients with cancer.

A potential limitation of the current study is that it would have been beneficial to know what type of and how much physical activity and exercise participants were doing during the follow-up period, and future studies should aim to find reliable and innovative strategies to measure this activity without placing too much burden on participants. Additionally, it is important to note that accelerometers are unable to record certain exercise modalities such as resistance exercise, cycling, or water sports. This may have resulted in an underestimation of PA, because of the device’s inability to capture activity performed during resistance exercise or cycling, which were the main components of the supervised exercise intervention. Strengths of this study include the long follow-up period, the comparison of two exercise regimens within the same trial, and the inclusion of both resistance and HIIT exercise in one of these groups.

### Clinical implications

A clinical implication from the current study is that participating in a targeted exercise program for women with breast cancer can confer long-term benefits on certain outcomes. We also showed that despite participation in these supervised exercise programs, and motivational support in the follow-up period, that patients may require greater support to maintain exercise levels. Patients with cancer and medical professionals [22] and recognise the well-known benefits of exercise, yet observational data show that patients are generally insufficiently physically active [23]. Strategies are needed to create pathways and support patients to exercise independently throughout the cancer continuum. Suggested approaches may include, but not be limited to, ensuring optimal symptom management [24], not only creating awareness of the value of exercise but, crucially, providing both patients and health professionals exercise resources and access to a qualified exercise specialist [24, 25], encouraging policy makers to fund exercise referral schemes for people with cancer where sufficient evidence for the benefits of exercise exists [26], and finally, individualising exercise prescriptions and behaviour change strategies to the needs and goals of the individual [26].

## Conclusions

Participants in the exercise groups of the OptiTrain RCT were generally still experiencing favourable differences in a number of physiological outcomes and symptoms to UC. Participants in the RT-HIIT group reported significantly lower total and cognitive cancer-related fatigue, and had higher leg muscle strength (reaching clinically meaningful effect sizes) than UC at 2 years. However, the AT-HIIT group reported significantly lower total symptoms and symptom burden, and body mass at 2 years post-baseline. While it is positive that the participants did not decrease their levels of MVPA from baseline to 2 years, only 3% of patients were meeting current physical activity guidelines (150 min of MVPA per week) at 2 years, indicating that this patient group may require significant support to reach and then maintain recommended levels of physical activity.

## Data Availability

The datasets used and/or analysed during the current study are available from the corresponding author on reasonable request.

## Abbreviations

1-RM: One repetition maximum
CI: Confidence intervals
CRF: Cancer-related fatigue (used in the tables only)
AT-HIIT: Concurrent aerobic high-intensity interval training and continuous moderate-intensity aerobic exercise training
EORTC-QLQ-C30: The European Organisation for Research and Treatment of Cancer quality of life questionnaire
ES: Effect size
FITT: Frequency, intensity, type, time
HIIT: High-intensity interval training
MSAS: Memorial symptom assessment scale
MVPA: Moderate to vigorous physical activity
PACT: The physical activity during Cancer Treatment study
PFS: Piper fatigue scale
PPT: Pressure pain threshold
QoL: Quality of life
RCT: Randomised controlled trial
RPE: Rating of perceived exertion
RT-HIIT: Concurrent aerobic high-intensity interval training and resistance exercise training
SD: Standard deviation
SED: Sedentary behaviour
UC: Usual care
